# Case Report: Bell’s palsy: a neurological manifestation of COVID-19 infection

**DOI:** 10.12688/f1000research.140514.2

**Published:** 2024-06-28

**Authors:** Amro Abdelrahman, Amira Bitar, Isra Babiker, Fawaz Elgak, Mohamed Elgassim

**Affiliations:** 1 Department of Medical Education, Hamad Medical Corporation, Doha, Doha, Qatar

**Keywords:** COVID-19, Coronavirus, Bell's palsy, Isolated facial neuropathy, Acute peripheral neuropathy

## Abstract

**Background:**

Coronavirus (COVID-19) is the causative agent of the most recent pandemic that hit the globe and has been the cause of a vast range of symptoms, including neurological symptoms. Bell’s palsy is an acute peripheral facial paralysis commonly associated with viral infections.

**Case presentation:**

This case report describes a patient with incidental COVID-19 infection that led to acute unilateral peripheral facial paralysis, Bell’s palsy. Our patient is a 35-year-old male with no known comorbidities who was presenting with upper respiratory tract infection symptoms and was found to be positive for COVID-19. Soon after the onset of symptoms, he also developed right-sided facial weakness in association with his symptoms. A thorough examination revealed a peripheral neurological lesion. The diagnosis of Bell’s palsy secondary to COVID-19 virus infection was through the exclusion of other possible causes.

**Conclusions:**

This case report suggests a potential link between Bell’s palsy and COVID-19, highlighting the importance of a comprehensive understanding of the neurological manifestations of COVID-19. Further research is essential to determine the significance of neuropathies in COVID-19 and enhance treatment strategies.

## Introduction

Coronavirus (COVID-19) infection has affected millions of people worldwide. It’s an infectious disease caused by the severe acute respiratory syndrome coronavirus 2 (SARS-CoV-2). People with COVID-19 infection have a wide range of reported symptoms, ranging from a mild cough to acute respiratory syndrome (ARDS). ^1^ Patients with COVID-19 commonly face complications and potential causes of mortality, including conditions such as sepsis, acute kidney injury, ARDS, acute hypoxic encephalopathy, and acute cardiac injury. ^1^

A growing number of COVID-19 cases have been associated with facial nerve paralysis, often presenting as the initial symptom or occurring within the first week after the onset of viral symptoms or a positive COVID-19 test. ^2^

The relationship between COVID-19 and neurological complications, including facial palsy, is complex and has been the subject of ongoing research. While some cases report facial nerve paralysis occurring within a week of viral symptom onset, there is variability in the reported timeframes. ^3^

Additionally, individuals may experience other neurological complications such as loss of smell (anosmia), altered taste perception (dysgeusia), encephalopathy, Guillain-Barre syndrome, Miller-Fisher syndrome, and polyneuritis cranialis. ^4^ In this case we’re reporting a case of Bell’s palsy following COVID-19 infection in a previously healthy 35-year-old-male. This case report follows the CARE guidelines. ^15^

## Case presentation

A 35-year-old South Asian male taxi driver with no past medical history presented to the Emergency Department of Hamad General Hospital complaining of a two day history of sudden right-sided facial weakness associated with fever, cough, and sore throat. Three days before admission the patient had gone to a primary health care center due to upper respiratory tract symptoms, and a diagnosis of COVID-19 was made.

Two days later, the patient suddenly developed weakness associated with numbness, drooling saliva while eating and difficulty closing the right eye. He had no other neurologic symptoms and denied ear pain, skin rash, or arthralgia. He had no recent history of travel, or a tick bite. The review of other systems was unremarkable.

At the Emergency Department, his temperature was 37°C, his peripheral pulse rate was 70 bpm, and his respiratory rate was 18 breaths per minute. His oxygen saturation was 98% on room air, blood pressure was 122/74 mmHg. Physical examination revealed the absence of right-sided forehead wrinkles compared to the left, drooping of the right eyelid, and prominent mouth deviation suggestive of right lower motor neuron facial nerve palsy. Careful examination of ears showed dry impacted wax in the right ear with no vesicles. Examination of the parotid gland was unremarkable. Sensation in both upper and lower extremities was intact. No weakness was noted in either the upper or lower limbs. Kernig’s and Brudzinski’s signs were negative. Examination of other systems was unremarkable.

The patient’s complete blood count and basic metabolic panel were within normal ranges. His COVID-19 rapid antigen test was positive. The chest X-ray was unremarkable. A diagnosis of Bell’s palsy potentially related to COVID-19 infection was made, and the patient was discharged and prescribed the following medications:

‐ Prednisolone: 20 mg orally (PO) daily for 10 days.
‐ Levocetirizine: 5 mg PO daily for 5 days.
‐ Gentamicin solution: 0.3% solution, applied twice daily to both eyes for 7 days.
‐ Paracetamol: 1,000 mg PO every 6 hours as needed for 5 days.
‐ Eye drops: Applied twice daily for 14 days.

He was also referred to a physical therapy clinic. On his four-week follow-up visit, the patient showed no significant improvement. On his 10-week follow up clinic appointment, the patient symptoms showed a significant improvement.

## Discussion

Besides the usual and well-known respiratory symptoms, SARS-CoV-2 can affect the peripheral and central nervous systems. Neurological symptoms can be the first manifestation of COVID-19 infection or concurrent respiratory symptoms. A retrospective review reported neurological symptoms in 36.5% of patients. ^5^

Two different mechanisms could explain the neuropathogenesis of SARS-CoV-2. The first mechanism is due to endothelial damage and the subsequent passing of the virus from the systemic circulation to the cerebral circulation. The alternative mechanism is thought to be due to the direct entering of the virus through the cribriform wall and olfactory bulb, where the olfactory nerve terminates. ^6^ Using the olfactory pathways, the virus can harm the central nervous system (CNS), which may propagate from neuron to neuron by axonal transport. ^7^

When glial cells get infected with the virus, the body enters a pro-inflammatory state and releases cytokines. The prolonged exposure to cytokines may lead to nerve damage. ^8^

Moreover, various types of neurological manifestations of COVID-19 infection have been reported. ^9^

An observational study conducted in Spain documented cases where COVID-19 was associated with cranial nerve manifestations. In one of these cases, polyneuritis cranialis developed on the third day, while in the other case, Miller Fisher syndrome occurred on the fifth day. ^10^ A previous study also described a case of an isolated facial paralysis presented after six days in a patient with COVID-19 infection. ^2^ Our patient experienced a lower motor neuron facial paralysis on the third day of his ongoing COVID-19 infection.

Bell’s palsy is a lower motor neuron impairment of the facial cranial nerve, manifesting acutely as a unilateral facial paralysis. ^11^ Although the reason for many cases is unidentifiable, the most common cause of peripheral facial palsy is attributed to infections, mainly Herpes Simplex Virus-1 (HSV-1), Varicella Zoster Virus (VZV), and Lyme disease. ^3^ Our patient denied any recent travel, trauma, insect bite, skin rash, joint pain, itchiness, or tingling sensation in the body. Physical examination was unremarkable, with no skin rash; the outer ear canal was clear, and no signs of meningitis. Causes such as autoimmune and vasculitis were excluded as the patient did not have any systemic findings. Human Immunodeficiency Virus (HIV) infection was also excluded as it is a part of the infectious screening for all people getting their residencies in the country. The patient had a fever, sore throat, and generalized body pain, and his COVID-19 rapid antigen test came positive. Therefore, no other etiologies than COVID-19 infection could be attributed to palsy.

COVID-19 infection is known to present mainly as respiratory symptoms ranging from mild to severe, such as ARDS and fever. ^12^ In addition, neurological manifestations, including Guillain-Barre syndrome, anosmia/ageusia, encephalopathy, and myelitis, are also encountered. ^3^ Bell’s palsy is one of the manifestations of COVID-19. Poor prognosis is predicted in patients >60 years of age with systemic problems such as diabetes mellitus, severe pain in the ear, and decreased tear production. Bell’s palsy generally has a good prognosis and recovery rate of 90%. ^13^ Regarding the treatment, the most used one in facial paralysis is corticosteroids, with high effectiveness rates. ^12^ Prednisolone’s effect on the facial nerve is by reducing its edema. ^14^ In our case, the patient had no risk factors for poor prognosis; he was prescribed prednisolone (20 mg) for 10 days and referred to a physiotherapy clinic. His four-week follow-up visit showed no significant change in his condition. However, on his 10-week follow-up clinic visit his weakness improved significantly.

### Limitations

The viral screening wasn’t done to rule out all other viral etiologies.

## Conclusions

This case report raises the possibility that Bell’s palsy and COVID-19 infection are related. However, to prove the causal association, more cases with epidemiological data are required. For a deeper knowledge of COVID-19 infection, it is crucial to investigate its neurologic symptoms. Therefore, additional research is needed to fully understand the prognostic importance of cranial neuropathies in COVID-19 disease and their natural history and choose the most effective therapy approach.

### Key clinical message

Any patient with a fever and neurological symptoms should be evaluated for COVID-19.

## Consent

Written informed consent for publication of their clinical details was obtained from the patient.

## Data Availability

All data underlying the results are available as part of the article and no additional source data are required. Zenodo: CARE checklist for “Case Report: Bell’s palsy: a neurological manifestation of COVID-19 infection”. https://doi.org/10.5281/zenodo.8359632. ^15^ Data are available under the terms of the Creative Commons Attribution 4.0 International license (CC-BY 4.0).

## References

[1] Chen T Wu D Chen H : Clinical characteristics of 113 deceased patients with coronavirus disease 2019: retrospective study [published correction appears in BMJ. 2020 Mar 31;368:m1295]. *BMJ.* 2020;368:m1091. Published 2020 Mar 26. 10.1136/bmj.m1091 32217556 PMC7190011

[2] Goh Y Beh DLL Makmur A : Pearls & Oy-sters: Facial nerve palsy in COVID-19 infection. *Neurology.* 2020;95(8):364–367. 10.1212/WNL.0000000000009863 32439822

[3] Lima MA Silva MTT Soares CN : Peripheral facial nerve palsy associated with COVID-19. *J Neurovirol.* 2020;26(6):941–944. 10.1007/s13365-020-00912-6 33006717 PMC7531061

[4] Zammit M Markey A Webb C : A rise in facial nerve palsies during the coronavirus disease 2019 pandemic [published online ahead of print, 2020 Oct 1]. *J Laryngol Otol.* 2020;1–4. 10.1017/S0022215120002121 32998780 PMC7542321

[5] Montalvan V Lee J Bueso T : Neurological manifestations of COVID-19 and other coronavirus infections: A systematic review. *Clin Neurol Neurosurg.* 2020;194:105921. 10.1016/j.clineuro.2020.105921 32422545 PMC7227498

[6] Acharya A Kevadiya BD Gendelman HE : SARS-CoV-2 Infection Leads to Neurological Dysfunction. *J Neuroimmune Pharmacol.* 2020;15(2):167–173. 10.1007/s11481-020-09924-9 32447746 PMC7244399

[7] Dubé M Le Coupanec A Wong AHM : Axonal Transport Enables Neuron-to-Neuron Propagation of Human Coronavirus OC43. *J Virol.* 2018;92(17):e00404–e00418. Published 2018 Aug 16. 10.1128/JVI.00404-18 29925652 PMC6096804

[8] Li Y Fu L Gonzales DM : Coronavirus neurovirulence correlates with the ability of the virus to induce pro-inflammatory cytokine signals from astrocytes and microglia. *J Virol.* 2004;78(7):3398–3406. 10.1128/jvi.78.7.3398-3406.2004 15016862 PMC371061

[9] Filatov A Sharma P Hindi F : Neurological Complications of Coronavirus Disease (COVID-19): Encephalopathy. *Cureus.* 2020;12(3):e7352. Published 2020 Mar 21. 10.7759/cureus.7352 32328364 PMC7170017

[10] Gutiérrez-Ortiz C Méndez-Guerrero A Rodrigo-Rey S : Miller Fisher syndrome and polyneuritis cranialis in COVID-19. *Neurology.* 2020;95(5):e601–e605. 10.1212/WNL.0000000000009619 32303650

[11] Egilmez OK Gündoğan ME Yılmaz MS : Can COVID-19 Cause Peripheral Facial Nerve Palsy? *SN Compr Clin Med.* 2021;3(8):1707–1713. 10.1007/s42399-021-00967-4 34056546 PMC8140315

[12] Oke IO Oladunjoye OO Oladunjoye AO : Bell’s Palsy as a Late Neurologic Manifestation of COVID-19 Infection. *Cureus.* 2021;13(3):e13881. Published 2021 Mar 14. 10.7759/cureus.13881 33868845 PMC8043567

[13] Çaytemel B Kılıç H Çomoğlu Ş : Approach to otolaryngology emergency in COVID-19 pandemic. *Tr-ENT.* 2020;30(1):24–36. 10.5606/Tr-ENT.2020.26817

[14] Piercy J : Bell’s Palsy. *The BMJ.* 2005. Published June 9. Accessed September 30, 2022. 10.1136/bmj.330.7504.1374 Reference Source 15947400 PMC558292

[15] Abdelrahman A : Case Report: Bell’s palsy: a neurological manifestation of COVID-19 infection.[Dataset]. *Zenodo.* 2023. 10.5281/zenodo.8359632 PMC11809692 39931161

