# Supplementary material for: Case Report: Bell’s palsy: a neurological manifestation of COVID-19 infection
Source: F1000Res. 2024 Jun 28;12:1355. Originally published 2023 Oct 18. [Version 2] doi: 10.12688/f1000research.140514.2 (PMC11809692; doi:10.12688/f1000research.140514.2)
Supplement: Peer review history

# Reviewer report: Reviewer response for version 2

**DOI:** 10.5256/f1000research.168370.r328968

**Published:** 2024-12-28

**Authors:** Josef Finsterer

**Affiliations:** 1 Neurology and Neurophysiology Center,, Vienna, Austria

F1000REs, Case Report: Bell’s palsy: a neurological manifestation of COVID-19 infection

 Abdelrahman et al report a single case of a 35yo male patient with SARS-CoV-2 infection that was complicated by unilateral facial palsy.

**Major points:**

 Facial palsy is a common complications of SC2 infection and vaccinations. This should be added to the limitations

**Minor points:**

 Do not use the term “Bell’s palsy”. It is reserved for idiopathic facial palsy.

Are enough details provided of any physical examination and diagnostic tests, treatment given and outcomes?

Yes

Is the case presented with sufficient detail to be useful for other practitioners?

Yes

Is sufficient discussion included of the importance of the findings and their relevance to future understanding of disease processes, diagnosis or treatment?

Yes

Is the background of the case’s history and progression described in sufficient detail?

Yes

Reviewer Expertise:

neuromuscular

I confirm that I have read this submission and believe that I have an appropriate level of expertise to confirm that it is of an acceptable scientific standard.

# Reviewer report: Reviewer response for version 2

**DOI:** 10.5256/f1000research.168370.r346701

**Published:** 2024-12-26

**Authors:** Isabel Vilaseca

**Affiliations:** 1 Universitat de Barcelona, Barcelona, Catalonia, Spain

In the treatment section, please explain why the patient required gantamicin in both eyes and levocetiricine. I cannot understand the rationale of this prescription.

 In the discussion a paragraph should be included to highlight that the association of Bell's palsy  and COVID-19 is still controversial.

 In fact, a recent historical cohort comparison study evaluating the crude incidence of patients diagnosed with Bell's palsy, did not find association between vaccination or COVID-19 infection and the development of Bell's Palsy before and after the pandemic period.

Are enough details provided of any physical examination and diagnostic tests, treatment given and outcomes?

Partly

Is the case presented with sufficient detail to be useful for other practitioners?

Yes

Is sufficient discussion included of the importance of the findings and their relevance to future understanding of disease processes, diagnosis or treatment?

Partly

Is the background of the case’s history and progression described in sufficient detail?

Yes

Reviewer Expertise:

Otorhinolaryngology

I confirm that I have read this submission and believe that I have an appropriate level of expertise to confirm that it is of an acceptable scientific standard, however I have significant reservations, as outlined above.

## References

[1] : Bell's palsy and COVID-19: A cohort study with historical rate comparison. *Acta Otorrinolaringol Esp (Engl Ed)* .2024; 10.1016/j.otoeng.2024.07.004 10.1016/j.otoeng.2024.07.004 39306084

# Reviewer report: Reviewer response for version 2

**DOI:** 10.5256/f1000research.168370.r296810

**Published:** 2024-07-16

**Authors:** Sohyeon Kim

**Affiliations:** 1 Department of Neurology, Keimyung University, Daegu, South Korea

I acknowledge some of the changes made by the authors and I respect the effort.

Are enough details provided of any physical examination and diagnostic tests, treatment given and outcomes?

Partly

Is the case presented with sufficient detail to be useful for other practitioners?

Partly

Is sufficient discussion included of the importance of the findings and their relevance to future understanding of disease processes, diagnosis or treatment?

Partly

Is the background of the case’s history and progression described in sufficient detail?

Partly

Reviewer Expertise:

neuromuscular diseases

I confirm that I have read this submission and believe that I have an appropriate level of expertise to confirm that it is of an acceptable scientific standard.

# Reviewer report: Reviewer response for version 1

**DOI:** 10.5256/f1000research.153880.r274184

**Published:** 2024-05-21

**Authors:** Sohyeon Kim

**Affiliations:** 1 Department of Neurology, Keimyung University, Daegu, South Korea

I found it as a quite interesting case, but there are several important issues to be checked over in this case report.

 First of all, the diagnostic process seemed somewhat insufficient to me. If someone wants to attribute a certain neurologic symptom to be caused by COVID-19, he/she should explain either a causative mechanism or a temporal relationship together with thorough exclusion of other causes.

 In the Case presentation section, the author stated as "A diagnosis of Bell's palsy secondary to COVID-19 infection was made". I think this sounds too confirmative since the causative mechanism of COVID-19 triggering Bell's palsy in this case is uncertain. Also, regarding the exclusion of other causes, the case is lacking of objective results from the diagnostic evaluations and the diagnosis mostly depended merely on the implications from clinical symptoms. If the authors had performed any laboratory test about autoimmune etiology or auxiliary diagnostic work up such as nerve conduction study, cranial MRI, and etc., I recommend containing the results in the revised manuscript. If the objective data are unavailable, the author should mention this as a limitation and need to tone down about the diagnostic certainty.  

 Secondly, In the Introduction section, the author mentioned "A growing number of COVID-19 cases have been associated with facial nerve paralysis, often presenting as the initial symptom or occurring within the first week after the onset of viral symptoms or a positive COVID-19 test". While I acknowledge that facial paralysis occurred within three days of the COVID-19 diagnosis in the present case and it is close enough to allege their temporal relationship, the above sentence sounds to me as if facial palsy and COVID-19 need to occur within one week from each other to generally define the temporal relationship. As there have been controversy among researchers regarding the definition of the time interval as evidence of a specific temporal relationship between COVID-19 and neurological complications, I suggest that the authors supplements the manuscript with additional references to clarify the definition of the temporal relationship between facial palsy and COVID-19.

 Also, I am not sure if we could adopt the same prognostic factors of Bell's palsy in COVID-19 related cases as the authors mentioned in Discussion section. I think the clinical characteristics and prognostic factors specifically of COVID-19 related Bell's palsy cases should be investigated and explained with supplementary references. 

 Additionally, there are several minor comments for the manuscript as well. 

 - In the Case presentation section, the phrase "no past medical history" is repeated in the first and second paragraph, and I recommend omitting one of them to avoid repetition.

 - In the Discussion section, I think it is out of context to mention the encephalopathy case in the fourth paragraph, or the paragraph can be improved with additional references as there are numerous case reports so far about diverse neurologic manifestations following COVID-19 infection.  

 The author presented with an intriguing cases for sure, but I think the manuscript needs a major revision.

Are enough details provided of any physical examination and diagnostic tests, treatment given and outcomes?

Partly

Is the case presented with sufficient detail to be useful for other practitioners?

Partly

Is sufficient discussion included of the importance of the findings and their relevance to future understanding of disease processes, diagnosis or treatment?

Partly

Is the background of the case’s history and progression described in sufficient detail?

Partly

Reviewer Expertise:

neuromuscular diseases

I confirm that I have read this submission and believe that I have an appropriate level of expertise to confirm that it is of an acceptable scientific standard, however I have significant reservations, as outlined above.

# Response

**Published:** 2024-06-24

**Authors:** Amro Abdelrahman

Dear reviewer 

 Thanks for taking time and review our article.

 - The diagnosis of Bell's palsy is primarily clinical and typically idiopathic. Our diagnosis was based on the acute presentation of classical symptoms, alongside a recent history of upper respiratory infection. According to local protocols, additional investigations such as nerve conduction studies, MRI, or CT are not routinely indicated unless systemic manifestations, recurrent symptoms, or other nerve involvement are present. Further investigations may be warranted in cases with atypical features.

 - I agree with you we're not 100% sure about COVID-19 being  the etiology in this case. I think changing the sentence to "A diagnosis of Bell's palsy potenially related to COVID-19 infection was made" would be suffecient.

 - The introduction section, a new sentence with a reference will be added for clarification "The relationship between COVID-19 and neurological complications, including facial palsy, is complex and has been the subject of ongoing research. While some cases report facial nerve paralysis occurring within a week of viral symptom onset, there is variability in the reported timeframes."

 - We just highlighted briefly some of the poor prognostic factors for the healthcare providers to give extra attention and close follow up for those with the mentioned poor prognostic factors. We believe (prognostic factors in bells palsy related to COVID-19) is a big topic to be included in a case report and need a different paper to extensively go through it. 

 - Past medical history will be deleted in the second paragraph

 - The sentence will be rephrased to "Various types of neurological manifestations of COVID-19 infection have been reported."  with a new referance
